# Safety and Efficacy of Adjunctive Heparin Infusion During Catheter-Directed Thrombolysis for Acute Limb Ischemia

**DOI:** 10.7759/cureus.101913

**Published:** 2026-01-20

**Authors:** Ahmed Mehanna, Sonia Franchini, Ayman Zyada, Gabriel Sayer

**Affiliations:** 1 Vascular Surgery, University Hospitals of Leicester NHS Trust, Leicester, GBR; 2 Vascular Surgery, St George’s University Hospital, London , GBR; 3 Vascular Surgery, Aberdeen Royal Infirmary, Aberdeen, GBR; 4 Vascular Surgery, Barking, Havering, and Redbridge University Hospitals, London, GBR

**Keywords:** acute limb ischemia, bleeding complications, catheter-directed thrombolysis, endovascular thrombolysis, heparin, intra-arterial thrombolysis, limb salvage, thrombosis

## Abstract

Background

Acute limb ischemia (ALI) requires urgent revascularization to prevent limb loss and mortality. Catheter-directed thrombolysis (CDT) is an established treatment; however, the role of concomitant intra-arterial heparin infusion during CDT remains unclear. Current guidelines discourage routine therapeutic heparinization, yet clinical practice varies. This study evaluated the efficacy and safety of adjunctive heparin infusion during CDT for ALI.

Methodology

A retrospective single-center study was conducted, including all patients who underwent CDT for ALI between November 2022 and May 2024. Patients were categorized into two groups: group 1 received intra-arterial heparin infusion during CDT, whereas group 2 underwent CDT without adjunctive heparin. Outcomes included procedural success (defined as thrombus resolution, limb salvage, and absence of additional revascularization) and complications (death, International Society on Thrombosis and Hemostasis (ISTH)-defined major bleeding, and minor bleeding). Statistical analysis was performed using SPSS version 27 (IBM Corp., Armonk, NY), with significance set at *P* < 0.05.

Results

Eighty-one patients were included, with 43 in group 1 and 38 in group 2. Procedural success rates were similar between the groups (74.4% vs. 78.9%, *P* = 0.632). However, overall complications were significantly higher in group 1 (19/43, 44.2%) compared with group 2 (8/38, 21.1%; *P* = 0.030). Group 1 had more minor bleeding events (15/43, 34.9%) compared with group 2 (8/38, 21.1%) and accounted for all major bleeding (1/43, 2.3%) and mortality events (2/43, 4.7%). Thrombolysis duration was significantly longer in group 1 (median 28 vs. 15 hours, *P* = 0.001).

Conclusions

Concomitant intra-arterial heparin infusion during CDT does not improve procedural success and is associated with higher complication rates, particularly bleeding. These findings support current guideline recommendations against routine heparin infusion during CDT and highlight the need for further prospective research to optimize anticoagulation strategies in ALI management.

## Introduction

Acute limb ischemia (ALI) is a vascular emergency characterized by a sudden reduction in arterial blood flow to a limb, leading to an imminent threat to tissue viability. Prompt recognition and intervention are critical, as delayed management can result in irreversible tissue damage, limb loss, or even death. According to the European Society for Vascular Surgery (ESVS), ALI is diagnosed when symptoms, such as pain, pallor, pulselessness, paresthesia, and paralysis, persist for less than two weeks; if the ischemic symptoms extend beyond this timeframe, the condition is typically classified as chronic limb-threatening ischemia (CLTI) [1].

The etiology of ALI is often categorized as embolic, thrombotic, or due to in situ thrombosis of an atherosclerotic artery or bypass graft. Embolic events frequently originate from cardiac sources, such as atrial fibrillation, whereas thrombotic occlusions tend to arise in patients with pre-existing peripheral artery disease. Identifying the underlying cause is essential, as it influences both acute management and long-term therapeutic strategies.

Catheter-directed thrombolysis (CDT) has emerged as a pivotal endovascular intervention for ALI, particularly in patients with threatened limb viability but without immediate indications for surgical revascularization. CDT involves the localized administration of fibrinolytic agents directly into or adjacent to the thrombus via arterial access, most commonly through the femoral artery. Recombinant tissue plasminogen activator (rtPA) and urokinase are the most widely used thrombolytics, given their proven efficacy in lysing arterial thrombi while minimizing systemic fibrinolytic exposure [2].

CDT is especially indicated in patients with Rutherford category IIa and IIb ischemia, where there is a viable or marginally threatened limb, respectively. In such cases, CDT can restore perfusion without the need for open surgical thrombectomy, thereby reducing perioperative morbidity. Furthermore, CDT has demonstrated effectiveness in the recanalization of acutely thrombosed bypass grafts, which are often challenging to manage surgically [2,3].

Despite widespread clinical practice, the role of concurrent anticoagulation with heparin during CDT remains controversial. Current ESVS guidelines recommend against routine continuous systemic therapeutic heparinization during thrombolytic therapy, citing the lack of robust evidence for improved outcomes and the potential for increased bleeding risk [3]. Nonetheless, many clinicians administer low-dose unfractionated heparin (UFH) through the introducer sheath to maintain catheter patency and reduce the risk of pericatheter thrombosis. This practice is supported primarily by observational data and institutional experience rather than controlled studies [4].

Similarly, the use of low-molecular-weight heparin (LMWH) during CDT has not been shown to confer any measurable clinical benefit in the context of limb salvage or thrombus resolution. Consequently, the decision to use anticoagulation adjunctively with CDT often varies between centers, reflecting a lack of consensus and highlighting the need for further investigation.

Given these uncertainties, there is a critical need to evaluate both the efficacy and safety of intra-arterial heparin infusion when administered concurrently with CDT. Understanding whether low-dose heparin improves catheter patency, thrombolysis rates, or limb salvage outcomes, without increasing the risk of bleeding or other complications, could inform standardized protocols and optimize patient care in ALI management. The present study aims to address this knowledge gap and provide evidence to guide clinical decision-making regarding anticoagulation strategies during CDT by assessing the procedural success and complication rates of CDT with and without heparin intra-arterial infusion.

## Materials and methods

This retrospective, single-center study evaluated patients presenting with ALI who underwent CDT between November 2022 and May 2024, a total period of 18 months. The medical records used in this study were de-identified before analysis, in accordance with ethical and institutional guidelines. All consecutive patients treated for ALI with CDT during this interval were included, while those with incomplete clinical documentation or undergoing thrombolysis for indications other than ALI were excluded. Patients were divided into two groups based on peri-procedural management: group 1 consisted of individuals who received concomitant intra-arterial unfractionated heparin infusion through the arterial sheath during CDT, whereas group 2 comprised those who underwent CDT without adjunctive heparin.

Postoperative follow-up was standardized and conducted at two intervals: two weeks in the acute-care (*hot*) clinic and six weeks in the routine outpatient clinic. The primary outcome was procedural success, defined as resolution of thrombus with limb salvage and no requirement for further revascularization procedures. Secondary outcomes included complications such as mortality, major bleeding, and minor bleeding. Major bleeding was defined according to International Society on Thrombosis and Haemostasis (ISTH) criteria as clinically overt bleeding that was fatal, involved a critical anatomical site, caused a hemoglobin decrease of at least 2 g/dL, or required transfusion of two or more units of red blood cells [5]. Minor bleeding was defined as clinically evident bleeding not meeting ISTH major criteria, including access-site or sheath-related hematoma.

Data analysis was conducted by the IBM SPSS Statistics version 27 software (IBM Corp., Armonk, NY). The Kolmogorov-Smirnov test was used to assess the normality of continuous variables, where normally distributed data were represented by mean ± standard deviation (SD) and compared by an independent t-test, while nonparametric data were represented by median and interquartile range (IQR) and compared by the Mann-Whitney U test. Categorical variables were represented by numbers and percentages and compared by the chi-square (χ2) test. Odds ratio and confidence interval were calculated to assess the outcome. Significance was considered at *P* < 0.05.

## Results

A total of 81 patients underwent CDT for ALI during the study period. Of these, 43 patients were assigned to group 1 (those who received concomitant intra-arterial heparin infusion), and 38 patients were assigned to group 2 (those who underwent thrombolysis without adjunctive heparin).

Complication rates differed between the two groups. In group 1, there were two deaths (2/43, 4.7%), one major bleeding event (1/43, 2.3%), and 15 minor bleeding events (15/43, 34.9%), resulting in 19 patients with complications (19/43, 44.2%). In group 2, no deaths (0/38, 0%) or major bleeding events (0/38, 0%) occurred, while eight minor bleeding events (8/38, 21.1%) were recorded, resulting in eight patients with complications (8/38, 21.1%). Patients without complications were 24/43 (55.8%) in group 1 and 30/38 (78.9%) in group 2.

Both deaths occurred in group 1; neither was directly attributable to intracranial or catastrophic bleeding. One patient died from multi-organ failure related to severe ALI and associated comorbidities, while the second died from cardiovascular complications during hospitalization. The single major bleeding event was a hemorrhagic brain stroke, while minor bleeding events were predominantly access-site or sheath-related hematomas.

Regarding procedural outcomes, CDT was successful in 32 patients in group 1 and 30 patients in group 2. Non-successful procedures occurred in 11 patients in group 1 and eight patients in group 2. The results are shown in Table 1.

**Table 1:** Summary of the results.

Outcome	Group 1 (Heparin, *n* = 43)	Group 2 (No heparin, *n* = 38)
Death	2 (4.7%)	0 (0%)
Major bleeding	1 (2.3%)	0 (0%)
Minor bleeding	15 (34.9%)	8 (21.1%)
Any complication	18 (44.2%)	8 (21.1%)
No complications	24 (55.8%)	30 (78.9%)
Successful procedure	32 (74.4%)	30 (78.9%)
Unsuccessful procedure	11 (25.6%)	8 (21.1%)

Table 2 summarizes the comparative statistical analysis between patients who received adjunctive intra-arterial heparin infusion and those who did not during catheter-directed thrombolysis. The two groups were comparable in age, with no statistically significant difference observed (68.37 ± 12.04 vs. 67.24 ± 11.81 years, *t *= 0.427, *P *= 0.670). However, the duration of thrombolysis was significantly longer in the heparin infusion group, with a median treatment time of 28 hours (IQR 24-48) compared with 15 hours (IQR 5-24) in the non-infusion group (*U* = 396, *P *= 0.001). There was no significant difference between the groups regarding the use of an alteplase bolus (44.2% vs. 39.5%, X^2 ^= 0.184, *P *= 0.668). Procedural success rates were similar in both groups (74.4% vs. 78.9%, *X*^2 ^= 0.230, *P *= 0.631). In contrast, complication rates were significantly higher in the heparin group, with complications occurring in 44.2% of patients compared with 21.1% in the non-infusion group (X^2 ^= 4.858, *P *= 0.028), corresponding to an odds ratio of 2.97 (95% CI 1.11-7.95), indicating a nearly threefold increased risk of complications associated with adjunctive heparin infusion.

**Table 2:** Statistical analysis results. Data on age are represented as mean ± standard deviation (SD) and compared by independent t-test, while data on thrombolysis time were represented as median (interquartile range (IQR)) and compared by Mann-Whitney U test. Categorical data (Alteplase bolus and outcome) were represented as numbers and percentages and compared by the chi-square (χ2) test. *Significance was considered at *P* < 0.05.

Analysis	Heparin infusion (*n* = 43)	No infusion (*n* = 38)	*P*-value	OR (95% CI)
Age (years)	68.37 ± 12.04	67.24 ± 11.81	0.670	NA
Time of thrombolysis (hours)	28 (24-48)	15 (5-24)	0.001*	
Standard (no Alteplase bolus)	24 (55.8%)	23 (60.5%)	0.668	
Alteplase Bolus	19 (44.2%)	15 (39.5%)		
Successful	32 (74.4%)	30 (78.9%)	0.631	1.29 (0.46-3.64)
Unsuccessful	11 (25.6%)	8 (21.1%)		
No complications	24 (55.8%)	30 (78.9%)	0.028*	2.97 (1.11-7.95)
Complications	19 (44.2%)	8 (21.1%)		

## Discussion

This study investigated the impact of concomitant intra-arterial heparin infusion during CDT for ALI, an area where clinical practice varies considerably despite limited supporting evidence. Existing literature aligns with the ESVS guidelines, which advise against continuous systemic therapeutic heparinization during CDT due to concerns about bleeding risk and a lack of demonstrated clinical benefit [3]. Nevertheless, several observational reports describe the use of low-dose unfractionated heparin infused through the sheath to preserve catheter patency, though these studies have been small and non-comparative, leaving uncertainty regarding its true efficacy and safety [4].

Braithwaite and Quiñones-Baldrich reported that heparin can be administered through the side arm of the arterial sheath or the outer catheter to reduce the risk of pericatheter thrombosis during CDT, particularly when the catheter is positioned antegradely within the vessel’s proximal flow [6]. Although a 2016 study evaluated outcomes of CDT with simultaneous intravenous unfractionated heparin infusion (500 IU/hour) despite ESVS guideline recommendations [7], more recent evidence from 2022 demonstrated that additional intravenous heparin during thrombolysis did not significantly impact re-occlusion rates or amputation-free survival, suggesting that adjunctive heparin may have limited influence on procedural outcomes in ALI [8]. Similar controversy has been reported in several other studies [9-14].

Our study contributes to this evidence gap by directly comparing outcomes in patients treated with CDT either with or without adjunctive heparin infusion. The findings of our study indicate that the addition of heparin infusion during CDT did not improve procedural success, as success rates were comparable between group 1 (74.4%) and group 2 (78.9%). These results are consistent with previous literature showing that successful thrombolysis is primarily influenced by factors such as clot burden, duration of ischemia, and fibrinolytic dosing rather than adjunctive anticoagulation strategies. More importantly, our data demonstrate a significantly higher complication rate in the heparin group. Patients receiving heparin infusion experienced more minor bleeding events, one major bleeding event, and two deaths, resulting in a complication rate nearly double that of the non-heparin group (44.2% vs 21.1%, *P* = 0.030). This supports concerns raised in previous studies regarding increased bleeding risks associated with unnecessary anticoagulation during thrombolysis. Additionally, the heparin group required a significantly longer duration of thrombolysis, which may itself predispose patients to complications and reflects the complex interplay between pharmacological and procedural factors.

These findings have meaningful implications for clinical practice. At present, the decision to administer concomitant heparin during CDT is largely based on clinician preference rather than evidence. This study suggests that routine intra-arterial heparin infusion does not confer clinical benefit and may increase the risk of adverse events. Reducing or eliminating its use could therefore improve patient safety, shorten thrombolysis duration, and align practice more closely with current guideline recommendations. Moreover, the results reinforce the importance of adhering to evidence-based thrombolysis protocols and highlight the need for further standardization of practice in vascular centers.

However, several limitations must be acknowledged. First, the retrospective design introduces potential selection bias and limits the ability to establish causality. Differences in operator preference or case complexity. Second, the study was conducted at a single center, which may limit generalizability to other institutions with different practices or patient populations. Finally, clinical characteristics such as Rutherford classification, cause of ALI, and comorbidities may influence outcomes.

Future research should seek to address these limitations through prospective, multicenter studies or randomized controlled trials designed to evaluate the role of adjunctive heparin during CDT in a controlled setting. Further investigation into optimal anticoagulation strategies may also be beneficial. Studies examining catheter patency, thrombus clearance dynamics, and biochemical markers of coagulation during CDT could provide important mechanistic insights. Ultimately, a clearer evidence base is needed to guide standardized protocols and reduce unwarranted practice variation.

## Conclusions

Our study adds to the growing body of evidence suggesting that concomitant intra-arterial heparin infusion during CDT does not improve procedural success and may increase the risk of complications. These findings support guideline recommendations and highlight the need for cautious use of adjunctive anticoagulation in the management of ALI.
